# Legitimacy in the ‘secular church’ of the United Nations

**DOI:** 10.1177/0047117820904094

**Published:** 2020-02-04

**Authors:** Jodok Troy

**Affiliations:** University of Innsbruck

**Keywords:** legitimacy, religion, UN charter, United Nations

## Abstract

This article argues that how the United Nations (UN) conceptualizes legitimacy is not only a matter of legalism or power politics. The UN’s conception of legitimacy also utilizes concepts, language and symbolism from the religious realm. Understanding the entanglement between political and religious concepts and the ways of their verbalization at the agential level sheds light on how legitimacy became to be acknowledged as an integral part of the UN and how it changes. At the constitutional level, the article examines phrases and ‘verbal symbols’, enshrined in the Charter of the ‘secular church’ UN. They evoke intrinsic legitimacy claims based on religious concepts and discourse such as hope and salvation. At the agential level, the article illustrates how the Secretary-General verbalizes those abstract constitutional principles of legitimacy. Religious language and symbolism in the constitutional framework and agential practice of the UN does not necessarily produce an exclusive form of legitimacy. This article shows, however, that legitimacy as nested in the UN’s constitutional setting cannot exist without religious templates because they remain a matter of a ‘cultural frame’.

Providing legitimacy is one the United Nations’ (UN) core functions. Existing research of conceptual accounts of legitimacy and issues of legitimation are often steered by a liberal framework that views legitimacy ‘as the product of aggregated consent’.^1^ Such a framework assumes that ‘contemporary power struggles and power relations generally operate outside any religio-ideological and ethical context’.^2^ Concepts of legitimacy, however, are not only the result of procedural, a-political matters. Concepts of legitimacy are also, in the words of Clifford Geertz, a matter of a ‘cultural frame’^3^ defining their meanings, values and purpose. Yet in the literature, there is a gap when it comes to the question how religious concepts and language contribute to the UN’s seemingly secular concept of legitimacy as enshrined in its constitutional set-up, other than potential drivers for delegitimation.^4^ Some scholars even suggest that the UN is a ‘secular Church’ which embodies a ‘repository of the community’s collective beliefs’^5^ which are inevitably a matter of a cultural and religious frame.

In this article, I argue that there is in fact a religious dimension of the UN’s conception of legitimacy. Although seen as a modern rational and secular institution, the UN’s concept of legitimacy has drawn to a significant degree on religious thought, images and language in its development and presentation of legitimacy. This account of the UN’s legitimacy lends itself to different interpretations, either leading to a normative argument or acknowledging the religious dimension but dismissing it as rhetoric. Although not abandoning these interpretations, the main thrust of this article is still another one. The UN’s legitimacy cannot exist without religious concepts, however offhanded they might have landed in the organization and however instrumentalized they are for rhetorical use.

My exploration of religious interventions in the concept of legitimacy within the ‘secular church’ UN shows ‘how concepts of and related to “religion” have emerged, altered, ebbed and flowed in relation to specific cultural and geographic histories’.^6^ Pointing out religious dimensions and interventions in secular discourses and the constitutional set-up of the UN is not to argue that there is a religious discourse in a productive sense responsible for the UN’s concept of legitimacy. The evidence available of constitutional adoptions (in the Charter) and agential adoptions (by the Secretaries-General) of religious concepts and language might as well be rhetorical. Hence, the article does not seek to overturn established arguments on power politics or notions of empire as organizational agendas.^7^ Moreover, the religious dimension of the UN does not necessarily produce a certain form of legitimacy. Rather, I suggest that legitimacy as nested in the UN’s constitutional setting and interpreted by its agents cannot exist without religious templates because any form of legitimacy takes place in a ‘symbolic cultural and value laden frame of reference’.^8^ This frame of reference, provided by the religious dimension of the UN’s concept of legitimacy, not only leads to normative and theoretical consequences. There are also practical consequences that I point out at the example of how the concept of human dignity reflects conceptions of rights and their enforcement.

After an introductory section on the concept and definition of legitimacy, I illustrate that the UN Charter consists of a constitutional entanglement between political and religious concepts as commonly ingrained in assumed secular political constructs. This section takes stock of the mutual constitution of religion, politics and the formal set-up of international organizations.^9^ Second, I argue, that zooming in on this entanglement offers an additional explanation on how legitimacy became an integral part of the UN. The constitutional entanglement between religious and political concepts and their discourses is interesting to reveal because it develops in the space between normative and empirical aspirations of legitimacy. For example, symbolic phrases or ‘verbal symbols’ enshrined in the Charter evoke and augment legitimacy claims, charged by religious concepts such as hope and salvation.^10^ Moreover, those legitimacy claims were present in the organization even before its members practised international political conduct in and via the organization.^11^ They might have played a minor role in the set-up of the organization and its concept of legitimacy and they continue to be used rhetorical. Considering the interrelation between politics, law and religion, however, these concepts have the power to influence current and future considerations of legitimacy and play a part in how the organization faces future challenges as the remainder of the article outlines. The religious dimension, for example, illustrates that the quest to limit the use of force for the sake of peace was a motivation to create the UN in the first place and for which the organization claimed legitimacy.^12^ At the same time, recognizing the religious dimension helps to understand today’s contestations over this originally limited claim.

## The UN’s legitimacy: secularized but religious

Research on political legitimacy (e.g. for international organizations or institutions of global governance) typically focuses on the sources of legitimacy, the practices of legitimation and delegitimation (in relation to actors), and the consequences of legitimacy.^13^ Research addressing the sources, practices and consequences of legitimacy frame issues of legitimacy either as an empirical question or, less often, as a normative question or concept.^14^ In political practice, however, there is a constant temptation to equate legitimacy with legal or moral justifications for political conduct,^15^ questioning a clear-cut distinction between empirical facts and normative assumptions. The temptation to equate legal, moral and political aspects of legitimacy increases the enforcement of legitimacy as a source of (legal) rule compliance.^16^ Like political practice that tends to conflate legitimacy with moral and legal righteousness, empirical research on legitimacy, its legal sources and practices tends to conflate ‘lawfulness’ and ‘rightness’,^17^ seeking a privileged moral position beyond politics. Such a take on legitimacy misses that legitimacy is not only the result of procedural matters but also the result of political judgement. Political judgements and processes, however, are inevitable matters of ‘cultural frames’.^18^ In other words, the world is not as demystified and a-political as commonly assumed. ‘The extraordinary’, in the words of Geertz, ‘has not gone out of modern politics, however much the banal may have entered; power not only still intoxicates, it still exalts’^19^ – and so does legitimacy because it is a matter of power and a matter of ‘the extraordinary’.

Legitimacy, as I take it here, refers ‘to an actor’s normative belief that a rule or institution ought to be obeyed. It is a subjective quality, relational between actor and institution, and is defined by the actor’s *perception* of the institution’. The ‘substance of the rule or from the procedure or source by which it was constituted’^20^ propels the actor’s perception of the institution. This is where this article sets in, identifying religious ‘substances’ (sources) and ‘procedures’ (practices) that constituted the UN’s legitimacy and its significance for political conduct (consequences) which are the result of political judgements. This framework acknowledges that politics is also a struggle over symbols, their sources and the right to use them^21^ under a cultural and thus religious frame. Symbols are frequently invoked narratives where:

different sources of legitimacy are crafted together in a narrative that binds the authority back to the impartial pursuit of the social purpose. This bundling of different sources of legitimacy leads to *legitimation narratives or strategies.* . . . Legitimation narratives usually do not consist of single claims or references, but rather a combination of different sources.^22^

Lamenting the lack of a comprehensive intellectual history of the UN, scholars point out that any approach towards this limitation ‘should attempt to trace the ideas that an organization has identified’.^23^ Often, those ideas served as sources of legitimacy narratives. Understanding the thrust of legitimacy’s sources, practices and consequences from an angle of their religious dimensions helps to illustrate the continuities and contingencies of the organization and the ideas it represents and seeks to enforce. This perspective also helps to disentangle the confusing mix between legitimacy and morality and their rival interpretations, often left unaddressed by International Relations perspectives of the UN, historical accounts of the UN and the ideas this organization has identified. Questions inquiring into the nature of the substances or procedures by which conceptualizations of legitimacy are constituted are thus not without interest for an analysis of how the UN evolved as a major guarantor of legitimacy and how this role is changing today.

In an assumed ‘secular age’,^24^ enlightened reason seems to rule the world. Such a perception misses the impact of religious ideas and norms on identity formation and political concepts. Secularism remains the prevailing paradigm, determining how we think about religion in politics that is individual, irrational and thus irrelevant.^25^ Certainly, ‘religion’ is too broad a category to operationalize in any meaningful way. Here, I illustrate that ‘religion’ and religious concepts need interpretation in particular contexts such as the circumstances and configurations of power.^26^ Such an approach is at odds with other ways of conceptualizing legitimacy, as either a moral right or a source of self-interest.^27^ This approach of interpreting religion and religious concepts derives from the assumption that ‘the international is better conceptualized as a terrain in which religious and political entanglements should be expected to generate creative, dynamic, and hybrid modes of social and political agency’.^28^ Ignoring ‘the relational dimensions of religion and international politics’, then, ‘encourages neglect of key moments in the production of religious and political identities and practices, as well as the implicit normative position taken by doing so’.^29^

While the UN provides procedural rules (e.g. international law), it also prescribes itself substantial concepts of legitimacy (e.g. human dignity) and agential practices (e.g. of the Secretary-General) to enact this legitimacy. Nonetheless, the UN’s protracted history and near universal membership is unique because it has led the organization to ‘represent the preponderant opinion of the foreign offices and other participants in the management of foreign affairs of the governments of Member States’.^30^ This universal aspiration of the UN resembles more of a constitutional character than a contract-based character. As I illustrate below, the Preamble and the body of the Charter point towards what others have described as an ‘ongoing process of interaction and not simply a substantive set of rules’.^31^ This interaction is open for change, particularly because of its religious sources. The following identifies religious sources (e.g. the belief in serving a higher moral purpose) of the UN’s concept of legitimacy, its (agential) practices (e.g. a dynamic interpretation of the Charter advocated by the Secretaries-General), and theoretical and practical consequences (e.g. the interrelation of power, religion and law). Eventually, this analysis also helps to explore why and how the transforming conceptualization of legitimacy affects its provision, particularly in terms of the practice of international law.

## Religious sources and practices of the UN’s legitimacy

Religious sources, concepts and language support the UN’s constitutional concepts of legitimacy and its aspiration of collective legitimation. The symbolic value of the language in the Charter and in particular its Preamble illustrates how politics is a struggle over political and religious concepts and the right to use them. In other words, the distinction between a normative and an empirical concept of legitimacy is fragile. This fragility becomes obvious when looking at the promises put forward in the Charter. In particular, a normative take on the UN often frames it as the aspiration of a ‘parliament of man’,^32^ seeking to promote democracy, informed by a belief in progress and modernization. In this framing, the constitutional setting of the UN symbolizes the belief in a higher power and a larger moral purpose.^33^ Yet it is a religious hope that governs this larger moral purpose, put forward in the Preamble. Tellingly, this kind of hope is hard-wired into every religion.^34^ The stress on hope is present in the first lines of the Preamble that seek to ‘reaffirm faith in fundamental human rights’ and ‘in the dignity and worth of the human person’.

Certainly, the UN’s Charter does not encompass obvious and unequivocal references to ‘God’ or religion. However, the Preamble states that the ‘nations express their faith in the dignity and worth of the human person’. We may live in a ‘secular age’ but the values, ideas and norms prohibited by secularism continue to influence how we live in the global age, because they provide substantial and procedural concepts of meaning. The picture of the ‘organizational repository of the community’s beliefs’ of the UN is thus intriguing as it also accounts for the ‘translation’ of religious semantics into political concepts of legitimacy.^35^ The Preamble sets out to reaffirm faith in the dignity of the human person and the concept of human rights to garner legal legitimacy. In 1948, the Universal Declaration of Human Rights, breeding an even stronger entanglement between political and religious conceptions of legitimacy, reinforced this aspect of legitimacy.^36^ As it is, however, the Charter remains an ambiguous document. On one side, it stresses human rights and freedom for all, on the other side, its main principle remains the equality of sovereign nations. The permeating reinforcement of legitimacy in human rights discourse implies that actors who seek legitimacy via the UN are responsible for upholding these values. Doing so, they acknowledge a societal conception of actors and agency who are themselves affected and influenced by religious concepts. The norms that support legitimacy, in other words, are ‘modern societies’ *own norms*’,^37^ much of them relying ‘heavily on the language of international law and appeal to universal moral values for its legitimation’.^38^ As such, they cannot be interpreted without a cultural frame, that is they can not be interpreted a-political without a cultural frame. Rather, processes of the legitimation of power involves the participating agents ‘in a web of reciprocal rights and obligations’ which are ‘moral concepts’ and ‘belong to the realm of culture, ideology and values’.^39^

The Charter’s Preamble forcefully puts forward these ‘moral concepts’ and their web of rights and obligations. This is especially because the Preamble manifestly transcribes the idea of translating religious semantics into political concepts of legitimacy. Commonly, the perception of the Charter is one that includes a larger and more open-ended group than the signatories themselves.^40^ Addressing both the writers and the audience of the document as ‘We the peoples’, the Preamble imagines itself as a document that includes all participating parties and, in particular, the human beings that ought to be served by the organization. It is thus crucial to notice the substantial vision and value of human beings that the Charter and in particular its Preamble put forward in the service of peace.^41^

The very existence of a Preamble is the work of the South African politician Jan Smuts. Smuts, due to his experience working with the League of Nations, proposed in the course of the 1945 San Francisco conference a Preamble to the Charter. Others considered his choice of entrance words, ‘The High Contracting Parties’, as too officious and bureaucratic. The famous choice of words, ‘We the Peoples’, is either the influence of the Congressman Sol Bloom or the academic Virginia Gildersleeve, both US delegates to the San Francisco conference.^42^ Essentially, these lines point to the organization’s endeavours for collective legitimacy. In the retrospective horror and ‘untold sorrows’ of the previous World Wars that many of the drafters experienced,^43^ the endeavours to set up an organization providing collective legitimacy to limit the use of force are a meaningful sacrifice for peace. As the following illustrates, the religious dimension of legitimacy helps to explain the originally limited focus on peace, granted by the limitation of force.

The installation of a formal organization, the ‘United Nations’ (i.e. united against Nazism and the axis powers) wants to ‘reaffirm faith’ in human rights and values (e.g. ‘the dignity and worth of the human person’). However, as secularists would point out, to have faith likely means that there is no physical basis for the content of this faith.^44^ The phrase ‘the dignity and worth of the human person’ is thus intriguing as ‘dignity’ is a religious concept.^45^ The following reference to the ‘obligation arising from treaties’ relates to the first phrase (to ‘reaffirm faith’). This reference essentially frames the following covenant in the Charter’s main body as a bond between the participating parties, warranted by specific practices. A substantial framing of legitimacy in the Preamble accompanies the procedural concepts such as Articles 39 and 51. This substantial framing based on religious sources assures the participating parties that the subscription to the UN’s principles is worth to bind them in this organization and outlines specific practices towards this end.

The Preamble promises ‘to save succeeding generations from the scourge of war’. In doing so, it claims that the sacrifices made during the World Wars, preceding the foundation of the organization, were not useless. Indeed, the management of violence is a key function of political orders delimiting illegitimate and legitimate forms of violence.^46^ In this regard, the Preamble and the Charter constitute sources of substantial and procedural legitimacy concepts. However, it is the conflictual arena between universal aspirations and the preservation of legitimate sovereignty, which embeds those concepts of legitimacy. Within this tension, the organization presents itself as a manifestation of hope, as illustrated above, and as an instrument of peaceful action. The substance of legitimacy, the values and the administrative processes in the Charter are thus more than just a matter of secularist legalism or materialistic brute-fact power politics. Rather, the Charter outlines specific practices in its main body, often framed in a language that blends religious and secular concepts together. The phenomenon of merging or translating religious concepts into a secular constitutional language is, for example, evident when it comes to issues of legitimacy and responsibility. For example, pointing at ‘evil’ individuals or acts is a common practice of actors in search of metaphysical justifications and, ultimately, legitimation of political conduct. The forums offered by the UN are no exception to this practice. They illustrate how a secularized rhetorical use of ‘evil’ embroils legitimacy and morality together in powerful ways.^47^

Given its religious sources, the Preamble resembles Christian covenant theory because it frames the content, following in the main body of the Charter, as a substantial bond rather than only as a functional (legal) contract.^48^ Two cases in point are the Charter’s Articles 39 and 51. There ‘is no sovereign power in the United Nations; it has no capacity to protect the order of international law from an existential threat to its own existence. Its dissolution is unlikely to lead to a civil war among factions making competing claims to speak in the voice of a global sovereign’.^49^ The legal foundations of the UN, in this sense, represent an antipode, a ‘counter religion’,^50^ to Carl Schmitt’s conceptualization of political power. Schmitt referred to the capability of the state to call on the state of emergency, the power to act and to make decisions in extreme situations as the paramount characterization of the modern state. ‘Sovereign’, for Schmitt, ‘is he who decides on the exception’.^51^ Indeed, Article 39 on the right to collective security and Article 51 on the right to self-defence can be read as an Anti-Schmittian expression. For the founding members, a ‘collective and multilateral consensus was necessary for legitimacy’.^52^ Yet paradoxically, this legal foundation also reinforces the organization’s limited legitimacy claim based on its preservation of the sovereign power of the UN’s members.^53^

The section on consequences will turn back to this limited claim of legitimacy by pointing out that there are good reasons for this narrow framing that stresses the limitation of force for the sake of peace as the initial justification for the organization’s legitimacy. Despite its initial moralistic tone in the Preamble, the Charter remains realistic about international politics. The rule of law that the Charter seeks to provide is a product of social practice, established by and seeking to enforce shared understandings and background knowledge. This understanding of the rule of law to avoid unnecessary violence shapes the participant parties perceptions of international political conduct and, in particular, their perception of legitimacy the UN provides,^54^ focusing on peace, rather than justice or humanity.

Collective legitimacy, however, remains an ‘aspect of the verbal rather than the executive functioning of the United Nations’^55^ and thus a matter of agential practice. It is here, where the religious dimension of the UN’s legitimacy sources potentially wield an extending influence on practices. The figure of the UN’s chief administrative officer visibly displays a functioning that seeks to supplement the weak executive functioning of his or her organization. Because of the language employed in its founding document, some characterized the Secretary-General, the UN’s chief administrative officer, as a ‘lay pope’.^56^ Equally so, others regard the organization as a ‘cathedral of the international community, the organizational repository of the community’s collective beliefs’.^57^ The Secretary-General has been an ‘influential participant in the legal discourse that influences world politics’.^58^ The practice of a dynamic interpretation of the Charter, based on the assumption that it serves a larger moral purpose, is a persistent tradition in international law and the conduct of international organizations. A dynamic interpretation is essentially a Christian approach to hermeneutics that seeks to focus on the ‘“spirit,” rather than the “letter,” of the law’.^59^ Hammarskjöld, for instance, captured the spirit of the Charter as something superior to the letter of the law in the following remarks: ‘The principles of the Charter are, by far, greater than the Organization in which they are embedded, and the aims which they are to safeguard are holier than the policies of any single nation or people’.^60^ Hammarskjöld identified in the first lines of the Preamble ‘the will of God’, meaning ‘that we should love our neighbour as ourselves’ and that we should ‘‘practice tolerance and live together in peace with one another as good neighbours’’.^61^

A reading of the Secretaries-General discourse on religious narratives to justify and bolster legitimacy claims by interpreting the Charter illustrates anticipations of post-secular transformations that influence international organizations and their agents. While dynamically interpreting the Charter, those agents potentially institutionalize the transformation of religious values and concepts such as human dignity. International organizations and their agents serve as a ‘normative transmission between differently situated international actors, whose principles originate from diverse cultural and ethical backgrounds, whether these are religious, secular, national, ethnic or cultural’.^62^ Several Secretaries-General ‘translated’ religious convictions into the institutional framework of the UN. In some cases, Secretaries-Genreal knowledge of religious and ethical values even informed their political practice.^63^ This is not surprising, as through informal practices and dynamic interpretations of the law, Secretaries-General convey more change in the UN and international law than through procedural concepts outlined in the Charter.^64^ Diplomats and international civil servants notions of serving peace even ‘predate[s] that of serving the prince’.^65^ Of course, such a notion of the Secretary-General’s role is an ideal-type. However, it is telling that this notion, encapsulated in the secular and sacred connotation of the Greek and Hebrew term of ‘messenger’, justifies a higher call by a sacred mission.^66^ In this sense, the majority of the Secretaries-General leaned towards a dynamic interpretation of the Charter and believed in a higher moral purpose of the organization, which, for them, fortify the UN’s aspirations for and provision of collective legitimacy.

For example, the first Secretary-General, Trygve Lie, expanded the role of his office into a more political one than set out by the Charter.^67^ He also emphasized the later often advocated relationship between peace and development; stressed the Secretary-General’s dedication to the Charter’s principles and highlighted the organization’s authority.^68^ Hammarskjöld’s legacy, among others, is one of preserving and expanding the integrity of the office.^69^ U Thant has been an ‘unsung’ mediator in the Cuban missile crisis.^70^ Boutros-Ghali became one of the most outspoken norm entrepreneurs for democracy while in office.^71^ Annan used various windows of opportunity to leverage structural change.^72^ The current Secretary-General, António Guterres, relies on his experience as UN High Commissioner for Refugees in the face of global movements to invoke the larger moral purpose of the organization.^73^ All this illustrates that the Secretary-General does not command much ‘hard’ power but has considerable power over the interpretation and verbalization of the organization’s principles.^74^ However, it is not the ‘lack of an army to command’, as Claude put it, ‘but [the] lack of a party to lead and a body politic to rally behind’.^75^

The Secretaries-General are themselves objects of contestations over legitimacy claims, as they ground their authority on the legitimacy of the perceived power they wield. The Secretary-General, at least, has the ‘constitutional licence to be as big a man as he can’.^76^ His interpretation and advocacy of this ‘constitutional licence’ also influences the discourse over the sources and practices of legitimacy. This is not to make a universal argument for all Secretaries-General or to over-generalize. Different Secretaries-General made different use of this potential to influence the discourse over legitimacy. However, it is to illustrate how religious sources potentially relate to political practices by providing meaning as the Secretaries-General link abstract concepts of legitimacy, a wider public and, at least assumed, global moral standards and values.

Indicating the Secretary-General’s global role, the Pope once said to him that ‘Vous êtes mon homologue laïque’.^77^ On similar grounds, Secretary-General Ban Ki-Moon ‘turned Pope Francis into a global moral leader and a living embodiment of the UN’s universal values’.^78^ For the pope and the Secretary-General alike,^79^ leadership requires a ‘bold and noble vision for the community’ of their respective constituencies. The Secretary-General must have the ‘elusive ability to make others connect emotionally and intellectually’ for ‘a larger cause that transcends their immediate self-interest’.^80^ In an age of an ever-growing ‘enlargement of human expectations’,^81^ in the words of Javier Pérez de Cuéllar, this is what is required in global political conduct where the UN has been framed as a ‘focal point of an emerging “world-oneness”’^82^ with the Secretary-General stylized as a vanguard of moral authority to warrant this legitimacy claim.^83^ In fact, the organization’s chief administrative officers tended to use their authority to challenge their organization’s original limited claim of legitimacy. They often acted as vanguards to identify new ‘ideas’ for the organization and served as ‘catalysts’^84^ for change. Authority, however, can be ‘legitimate only if those who exercise that authority are themselves perceived as legitimate’.^85^ An ever-growing ‘enlargement of human expectations’^86^ is what makes the UN and its leadership disputed but appealing, a status that maintains their presence as forces of providing legitimacy in world politics.

### The politics of legitimacy: human dignity and the quest for peace

So far, I have illustrated that there is a religious dimension of the UN’s concept of legitimacy, which is discernible in sources and practices. Nonetheless, the broader question remains what it means that the UN’s concept of legitimacy is more religious than common secular sense suggests. The religious dimension might as well be rhetorical. However, the foregoing analysis also encompasses consequences for the UN’s legitimacy that are more influential. As illustrated, legitimacy is not only a matter of procedures. It is also a matter of meaning which finds its expression in a ‘symbolic cultural and value laden frame of reference’.^87^ Legitimacy, like power, is neither desacralized nor demystified. Rather, matters of legitimation always carry with them ‘moral and ideological implication which are irreducible to formal rationality’.^88^ In the remainder of the article, I illustrate theoretical and practical consequences of this assessment by having a look at conceptualizations of international law and the unfolding of the concept of human dignity.

A major schism in conceptions of international law, according to Hurd, is between an ‘enchanted’ and a ‘disenchanted’ view of international law. ‘Enchantment’, as used by Hurd, is a ‘position which assumes that international law occupies a privileged political and moral position’. I outlined this view above at the UN’s turn to a restored language of morality since the end of the Second World War. Disenchantment, on the other side, ‘refers to an attitude toward international law and politics that does not include this prior commitment’.^89^ The enchanted view takes legalization as morally and politically progressive.^90^ In other words, the enchanted view is antipolitical, presenting ‘law as an alternative to politics as means for settling disagreements over what should be done; legal procedures and institutions are expected to take the place of power struggles’.^91^ A disenchanted view that focuses on politics, on the other side, assumes that power and law are interrelated. While the foregoing analysis illustrated that there is no escape from the relation between religion and politics, the analysis emphasized this take on the political quality of law and power. In modern societies, in particular, complexities ‘generate the problem of legitimation as a contestable ongoing process’.^92^ Power struggles marked by religious sources and practices influence procedural matters of providing legitimacy. The ramifications of this influence become even more obvious by having a look at the concept of human dignity.

The concept of human dignity, illustrated above as a religious source, might serve as a practical example of the religious dimensions’ consequences of the UN’s legitimacy. Certainly, often ‘the idea of dignity reflects sociohistorical conceptions of basic rights and freedoms’^93^ rather than generating them. This might as well have been the case when Gildersleeve added the very term ‘human dignity’ to the UN Charter’s Preamble.^94^ However, as illustrated, as a religiously connoted verbal symbol, human dignity is also a ‘philosophical statement’, implying ‘that rights are not derived from the state or any other external authority’.^95^ In the case of the UN, this vision, implemented by the discourse over humanitarianism, human rights and legalization came close to realizing ‘itself as an international executive agency for the world’.^96^

Providing collective legitimacy for the sake of peace is still a major function of the UN, but legitimating international justice and responsibility steadily takes over this function. Changes in interpreting the original limited concepts of legitimacy, the emphasis on individual agency, cultural dialogue and scholarship influence this development as well. For example, Non-Governmental Organizations were involved in the UN from the beginning. This development, often referred to as the ‘third UN’,^97^ also influenced the changing legitimizing role of the UN. The ‘third UN’ helped to garner legitimacy for newly arising nations, fading European Empires at the time or the framing of human rights as utopia, attributing them a sacred status.^98^ Taking an enchanted a-political view, the practices that helped garner legitimacy turned into a narrative of a linear progress, seeking a privileged moral position that emphasizes the quest for justice. Nonetheless, many of the newly identified and advocated ‘ideas’ that the ‘third UN’ entail, overlook that most of the dedications supported by the majority of the UN members are non-Western virtues. The dedications ‘pertain more to the matters of development and of rights and protection than to the matters of security, sovereignty and culture’,^99^ castigating those who seek to advance individual human rights.

The religious source of human dignity and the consequences it entails, offer a more realistic take on the UN’s place in the world rather than falling into the trap of legalization that replaces power struggles with legal principles and procedures. Often, the basis for legalization and humanitarianism’s optimism ‘is not an improvement in people’s lives but an improvement in human rights norms’.^100^ In a rather disenchanted view of legal measures, power and the quest of the UN, Secretary-General Ban Ki-Moon, for example, concluded that to take the quest for human dignity serious, change must come in political solutions. Humanitarian assistance alone ‘will never be the solution’.^101^ Such a view acknowledges that the legitimation for humanitarian assistance and political solutions takes serious that human ‘dignity involves a complex notion of the individual’.^102^ As such, human dignity has a ‘wide range of applications outside of the sphere of human rights’.^103^ Considering its religious dimensions, this is one future plight of the UN’s legitimacy. Realists, for example, have been aware of the power struggle over legitimacy and its symbolic thrust, other than marring it to an absolutist moral language and practice. Consequently, Realists do not see power and legitimacy as anti-ethical. They warned to equalize legitimacy and legality in political practice; as together, they evade the political character of international conduct.^104^ International politics, after all, is a struggle not only over power but also over legitimacy,^105^ which is itself a matter of meaning.

Providing legitimacy as outlined by the UN’s Charter is one way to restrain utter destruction in international politics as Realists feared during the heights of the Cold War and argued over with religious concepts of the Apocalypse themselves.^106^ While Realists articulated their fears with the help of religious concepts, the ‘secular church’ UN already imbued itself with them. As the foregoing analysis illustrates, this imbuement with religious concepts is likely because such an ambitious organization cannot do without turning to the religious realm in explaining and justifying itself and the legitimacy it seeks to provide. The internationalist humanitarian project of the twentieth and twenty-first centuries and the prevailing moral paradigm of international law might often be a ‘triumph of form over substance, legalism over reality, hope over experience’.^107^ Yet neither this pretended or real triumph nor its obvious failings can do without a religious dimension, which provides the meaning for both. After all, the UN remains a ‘secular *church*’ which, even if all other justifications fail, remains an embodiment of belief that seeks to legitimize the hopes it represents for a larger moral purpose.

## Conclusion

The UN includes more religious dimensions and resonances than existing research recognizes. This article illustrates that the UN, although seen as a modern secular and rational institution, in fact cannot maintain its legitimacy claims without religious templates. Illustrating this argument also shows the continuities and contingencies of the organization; how it shaped notions of legitimacy; and points out the fragile distinction between normative and empirical concepts of legitimacy. The religious dimension might as well has been thrown offhanded into the UN’s constitutional framework. The main point this article makes, however, is that in practice, the legitimacy of such a universal organization cannot exist without religious templates, however unintended this dimension might have been and often still is. Legitimacy is always also a political matter. As such, any form of legitimacy, particularly the one of a universal organization like the UN, takes place in a cultural frame of reference rather than outside any religious or ethical contexts. The illustration of this argument shows that the political power of the religious dimension affects future conceptualizations of legitimacy. The UN’s second Secretary-General was aware of his organization’s founding and operation on the grounds of realpolitik when he conceded that the UN was ‘not created in order to bring us to heaven, but in order to save us from hell’.^108^ Towards this end, however, he made it equally clear that the UN is not an organization just for the great powers, but also, perhaps mainly, ‘for all the others’.^109^ Arguing and practicing UN policies in this spirit requires taking serious the religious dimensions of the organization’s concept of legitimacy, as they remain deeply intermingled with politics.

## Notes

1. Amitai Etzioni, ‘On Communitarian and Global Sources of Legitimacy’, *The Review of Politics*, 73(1), 2011, p. 105.

2. Nikos Kokosalakis, ‘Legitimation Power and Religion in Modern Society’, *Sociological Analysis*, 46(4), 1985, p. 367.

3. Clifford Geertz, *Local Knowledge: Further Essays in Interpretative Anthropology* (New York: Basic Books, 1983), p. 143; Kokosalakis, ‘Legitimation Power and Religion in Modern Society’, p. 375.

4. For the take on religion as an enabler of delegitimation, see Michael Zürn, *A Theory of Global Governance: Authority, Legitimacy, and Contestation* (New York: Oxford University Press, 2018), p. 75.

5. On the ‘secular church’ see Joseph P. Lash, ‘Dag Hammarskjöld’s Conception of His Office’, *International Organization*, 16(3), 1962, p. 543. See also David Rieff, *A Bed for the Night: Humanitarianism in Crisis* (London: Vintage, 2002), pp. 276–7. On the ‘repository’ see Michael Barnett, ‘Bringing in the New World Order: Liberalism, Legitimacy, and the United Nations’, *World Politics*, 49, 1997, p. 541.

6. Erin K. Wilson, ‘Theorizing Religion as Politics in Postsecular International Relations’, *Politics, Religion & Ideology*, 15(3), 2014, p. 355; Michael Walzer, *Thinking Politically: Essays in Political Theory* (New Haven: Yale University Press, 2007), pp. 147–67.

7. Mark Mazower, *No Enchanted Palace* (Princeton, NJ: Princeton University Press, 2009); Mary Ann Glendon, *A World Made New: Eleanor Roosevelt and the Universal Declaration of Human Rights* (New York: Random House, 2001), Chapter 1; Stephen C. Schlesinger, *Act of Creation: The Founding of the United Nations: A Story of Superpowers, Secret Agents, Wartime Allies and Enemies, and Their Quest for a Peaceful World* (Boulder, CO: Westview Press, 2003); David L. Bosco, *Five to Rule Them All: The UN Security Council and the Making of the Modern World* (Oxford: Oxford University Press, 2009).

8. Kokosalakis, ‘Legitimation Power and Religion in Modern Society’, p. 368.

9. Helge Arsheim, ‘Religion’, in Jacob K. Cogan, Ian Hurd and Ian Johnstone (eds) *The Oxford Handbook of International Organizations* (Oxford: Oxford University Press, 2016), p. 493.

10. On a secular notion of those symbolic phrases, see Ian Hurd, ‘Legitimacy, Power, and the Symbolic Life of the UN Security Council’, *Global Governance*, 8(1), 2002, pp. 36–8; Thomas M. Franck, *The Power of Legitimacy Among Nations* (Oxford: Oxford University Press, 1990), pp. 91–110.

11. In a strict linguistic sense, phrases cannot be symbols. Here, however, I refer to phrases in the sense of ‘condensation symbols’ (or ‘verbal symbols’), which is ‘a name, phrase, or maxim which stirs vivid impressions involving the listener’s most basic values’. Doris Graber, *Verbal Behavior and Politics* (Urbana, IL: University of Illinois Press, 1976), p. 289.

12. On the permitting of the use of force for peace and security (rather than for justice), see Samuel Moyn, ‘The Universal Declaration of Human Rights of 1948 in the History of Cosmopolitanism’, *Critical Inquiry*, 40(4), 2014, p. 371.

13. Jonas Tallberg, Karin Bäckstrand and Jan A. Scholte, ‘Introduction: Legitimacy in Global Governance’, in Jonas Tallberg, Karin Bäckstrand and Jan A. Scholte (eds) *Legitimacy in Global Governance: Sources, Processes, and Consequences* (Oxford: Oxford University Press, 2018), pp. 4–5.

14. Tallberg et al., ‘Introduction’, pp. 8–9; Etzioni, ‘On Communitarian and Global Sources of Legitimacy’, pp. 105–6. Weber’s sources of legitimacy (tradition, charisma and legality), for instance, are descriptive. Max Weber, ‘Politics as a Vocation’, in Max Weber, Hans H. Gerth and C.W. Mills (eds) *From Max Weber: Essays in Sociology* (London: Routledge, 1991), pp. 77–128.

15. Inis L. Claude, ‘Collective Legitimization as a Political Function of the United Nations’, *International Organization*, 20(3), 1966, p. 368; Richard A. Falk, Mark Juergensmeyer and Vesselin Popovski (eds), *Legality and Legitimacy in Global Affairs* (Oxford: Oxford University Press, 2012); Rodney S. Barker, *Legitimating Identities: The Self-Presentations of Rulers and Subjects* (Cambridge: Cambridge University Press, 2001); Jennifer Gronau, ‘Signaling Legitimacy: Self-Legitimation by the G8 and the G20 in Times of Competitive Multilateralism’, *World Political Science*, 12(1), 2016, pp. 107–145.

16. Ian Hurd, *How to Do Things* with *International Law* (Princeton, NJ: Princeton University Press, 2017), p. 105.

17. Ian Hurd, ‘Enchanted and Disenchanted International Law’, *Global Policy*, 7(1), 2016, p. 98; Judith Nisse Shklar, *Legalism: Law, Morals, and Political Trials* (Cambridge: Harvard University Press, 1964).

18. Geertz, *Local Knowledge*, p. 143; see also Kokosalakis, ‘Legitimation Power and Religion in Modern Society’, p. 375.

19. Geertz, *Local Knowledge*, p. 143. Kantorowicz, in particular (referred to by Geertz) points out the relation of religious ideas and symbols to the secular world and the question of what constitutes modernity. Ernst Kantorowicz, *The King’s Two Bodies: A Study in Mediaeval Political Theology*, 7th ed. (Princeton, NJ: Princeton University Press, 1997).

20. Ian Hurd, *After Anarchy: Legitimacy and Power in the United Nations Security Council* (Princeton, NJ: Princeton University Press, 2007), p. 7; Ian Hurd, ‘Legitimacy and Authority in International Politics’, *International Organization*, 53(2), 1999, p. 387.

21. Hurd, ‘Legitimacy, Power, and the Symbolic Life of the UN Security Council’, pp. 36–8.

22. Zürn, *A Theory of Global Governance*, p. 70. Emphasis in original.

23. Thomas G. Weiss, ‘The United Nations: Before, During and After 1945’, *International Affairs*, 91(6), 2015, p. 1227; Thomas G. Weiss, Tatiana Carayannis and Richard Jolly, ‘The “Third” United Nations’, *Global Governance*, 15(1), 2009, p. 123; note 1.

24. Charles Taylor, *A Secular Age* (Cambridge: Belknap Press of Harvard University Press, 2007).

25. Wilson, ‘Theorizing Religion as Politics in Postsecular International Relations’, p. 349.

26. Cecelia Lynch, *Interpreting International Politics* (New York: Routledge, 2014), p. 91.

27. Allen E. Buchanan, *Justice, Legitimacy, and Self-Determination: Moral Foundations for International Law* (Oxford: Oxford University Press, 2007); Erik Voeten, ‘The Political Origins of the UN Security Council’s Ability to Legitimize the Use of Force’, *International Organization*, 59(3), 2005, pp. 527–57.

28. Jonathan C. Agensky, ‘Recognizing Religion: Politics, History, and the “Long 19th Century”’, *European Journal of International Relations*, 23, 2017, pp. 3–4.

29. Agensky, ‘Recognizing Religion’, pp. 21; 3–4.

30. Claude, ‘Collective Legitimization as a Political Function of the United Nations’, p. 372. See critically Ian Hurd, ‘Myths of Membership: The Politics of Legitimation in UN Security Council Reform’, *Global Governance*, 14, 2008, pp. 199–217. On the League of Nations, see Donald S. Birn, ‘The League of Nations Union and Collective Security’, *Journal of Contemporary History*, 9(3), 1974, pp. 131–159.

31. Matthew S. Weinert, *Making Human: World Order and the Global Governance of Human Dignity*, Configurations: Critical studies of world politics (Ann Arbor, MI: University of Michigan, 2015), p. 72.

32. Paul M. Kennedy, *The Parliament of Man: The United Nations and the Quest for World Government* (London: Allen Lane, 2006).

33. Guy Fiti Sinclair, ‘The International Civil Servant in Theory and Practice: Law, Morality, and Expertise’, *European Journal of International Law*, 26(3), 2015, p. 752.

34. Martin Riesebrodt, *The Promise of Salvation: A Theory of Religion* (Chicago, IL: University of Chicago Press, 2012).

35. Mariano P. Barbato, ‘Conceptions of the Self for Post-Secular Emancipation: Towards a Pilgrim’s Guide to Global Justice’, *Millennium*, 39(2), 2010, p. 550; Gregorio Bettiza and Filippo Dionigi, ‘How Do Religious Norms Diffuse? Institutional Translation and International Change in a Post-Secular World Society’, *European Journal of International Relations*, 21, 2015, pp. 621–46. I take the ‘translation’ of religious language and concepts into the secular realm as their translation into a ‘generally accessible language’. Jürgen Habermas, ‘Religion in the Public Sphere’, *European Journal of Philosophy*, 14(1), 2006, p. 10.

36. John Nurser, ‘The “Ecumenical Movement” Churches, “Global Order,” and Human Rights: 1938-1948’, *Human Rights Quarterly*, 25(4), 2003, pp. 841–81; Glendon, *A World Made New*; Miguel Vatter, ‘Politico-Theological Foundations of Universal Human Rights: The Case of Maritain’, *Social Research*, 80(1), 2013, pp. 233–260.

37. Chris Thornhill, ‘Political Legitimacy: A Theoretical Approach Between Facts and Norms’, *Constellations*, 18(2), 2011, p. 163, emphasis in original.

38. Mark Mazower, *Governing the World: The History of an Idea, 1815 to the Present* (New York: Penguin Books, 2013), p. 379.

39. Kokosalakis, ‘Legitimation Power and Religion in Modern Society’, p. 371.

40. Mika Luoma-aho, ‘Millenarian Development Goals: Commentary on the Political Theology of the Millennium Declaration’, in Péter Losonczi, Mika Luoma-aho and Aakash Singh (eds) *The Future of Political Theology: Religious and Theological Perspectives* (Farnham: Ashgate, 2011), p. 176.

41. Luoma-aho, ‘Millenarian Development Goals’, pp. 179–80; John Nurser, *For All Peoples and All Nations: The Ecumenical Church and Human Rights* (Washington, DC: Georgetown University Press, 2005).

42. Peter Marshall, ‘Smuts and the Preamble to the UN Charter’, *The Round Table*, 90(358), 2001, p. 57; Jean-Pierre Cot and Alain Pellet, ‘What They Had in Mind: The Preamble to the Charter’, *Development: Seeds of Change*, 1/2, 1986, p. 25; Schlesinger, *Act of Creation*, p. 237; Marshall, ‘Smuts and the Preamble to the UN Charter’, p. 59; Rosalind Rosenberg, ‘Virginia Gildersleeve: Opening the Gates’, *Living Legacies*, available at: https://goo.gl/qxWrTr.

43. Luoma-aho, ‘Millenarian Development Goals’, p. 181; Rosa Brooks, *How Everything Became War and the Military Became Everything: Tales From the Pentagon* (New York: Simon & Schuster, 2016), p. 191; Jebb Gladwyn, *The Memoirs of Lord Gladwyn* (New York: Weybright Talley, 1972), p. 94.

44. Luoma-aho, ‘Millenarian Development Goals’, pp. 175–6.

45. Hans Joas, *The Sacredness of the Person: A New Genealogy of Human Rights* (Washington, DC: Georgetown University Press, 2013).

46. Ian Hurd, ‘The Permissive Power of the Ban on War’, *European Journal of International Security*, 2(1), 2017, 1–18.

47. Anna Geis and Christopher Hobson, ‘The Existence and Use of “Evil” in International Politics’, *International Politics*, 51(4), 2014, p. 419; William D. Casebeer, ‘Knowing Evil When You See It: Uses for the Rhetoric of Evil in International Relations’, *International Relations*, 18(4), 2004, p. 442; Mona K. Sheikh, ‘Appointing Evil in International Relations’, *International Politics*, 51(4), 2014, pp. 492–507.

48. Luoma-aho, ‘Millenarian Development Goals’, pp. 181–82.

49. Paul W. Kahn, *Political Theology: Four New Chapters on the Concept of Sovereignty* (New York: Columbia University Press, 2012), p. 56.

50. Paul W. Kahn, *Sacred Violence: Torture, Terror, and Sovereignty* (Ann Arbor, MI: University of Michigan Press, 2008), p. 52.

51. Carl Schmitt, *Political Theology: Four Chapters on the Concept of Sovereignty* (Cambridge: MIT Press, 1985), p. 5.

52. Francis S. Fiorenza, ‘Prospects for Political Theology in the Face of Contemporary Challenges’, in Francis S. Fiorenza, Klaus Tanner and Michael Welker (eds) *Political* Theology: *Contemporary* Challenges *and* Future Directions (Louisville, KY: Westminster John Knox Press, 2013), p. 45.

53. Kahn, *Sacred Violence*, p. 54.

54. Hurd, *How to Do Things with International Law*, p. 53.

55. Claude, ‘Collective Legitimization as a Political Function of the United Nations’, p. 372.

56. Roger Lipsey, *Hammarskjöld: A Life* (Ann Arbor, MI: University of Michigan Press, 2013), p. 153. Hammarskjöld considered the UN as a ‘secular church’. Wilder Foote (ed.), *Dag Hammarskjöld: Servant of Peace* (New York: Harper & Row, 1962); A Selection of His Speeches and Statements, p. 47. As Hammarskjöld remarked, the UN stands ‘outside – necessary outside – all confessions but it is, nevertheless, an instrument of faith’. Foote, *Dag Hammarskjöld*, pp. 56–7.

57. Barnett, ‘Bringing in the New World Order’, p. 541.

58. Ian Johnstone, ‘The Role of the UN Secretary-General: The Power of Persuasion Based on Law’, *Global Governance*, 9(4), 2003, p. 441.

59. Fiti Sinclair, ‘The International Civil Servant in Theory and Practice’, pp. 752, 758; Weinert, *Making Human*, p. 72.

60. Security Council Official Records, *Eleventh Year, 751st Meeting* (1956), available at: http://goo.gl/yl1tyb, pp. 1–2; see also Javier Pérez de Cuéllar, *Pilgrimage for Peace: A Secretary-General’s Memoir* (Basingstoke: Macmillan, 1997), p. 16.

61. Foote, *Dag Hammarskjöld*, p. 56.

62. Bettiza and Dionigi, ‘How Do Religious Norms Diffuse?’, p. 3.

63. This is most obvious the case of Hammarskjöld. Dag Hammarskjöld, *Markings: Transl. From the Swedish by Leif Sjöberg & W. H. Auden* (New York: Vintage Spiritual Classics, 2006); Manuel Fröhlich, *Political Ethics and the United Nations: Dag Hammarskjöld as Secretary-General* (London: Routledge, 2008); Jodok Troy, ‘Dag Hammarskjöld: An International Civil Servant Uniting Mystics and Realistic Diplomatic Engagement’, *Diplomacy & Statecraft*, 21(3), 2010, pp. 434–50; Alynna J. Lyon, ‘The UN Charter, the New Testament, and the Psalms: The Moral Authority of Dag Hammarskjöld’, in Kent J. Kille (ed.) *The UN Secretary-General and Moral Authority*, (Washington, DC: Georgetown University Press, 2007), pp. 111–141; Filippo Dionigi, ‘Dag Hammarskjöld’s Religiosity and Norms Entrepreneurship: A Post-Secular Perspective’, *Politics and Religion*, 9(1), 2016, pp. 162–86.

64. G. Wiseman, ‘Diplomatic Practices at the United Nations’, *Cooperation and Conflict*, 50(3), 2015, pp. 316–33; Tapio Kanninen and Touko Piiparinen, ‘Why Bureaucracies Matter in the Global Age: A Post-Weberian Explanation with the Case Study of Preparing and Implementing the United Nations’ an Agenda for Peace’, *International Relations*, 28(1), 2014, pp. 46–66.

65. Paul Sharp, ‘Who Needs Diplomats? The Problem of Diplomatic Representation’, *International Journal*, 52, 1997, p. 616; Christer Jönsson and Martin Hall, *Essence of Diplomacy* (Houndmills: Palgrave Macmillan, 2005), pp. 116–7.

66. Sharp, ‘Who Needs Diplomats?’, p. 615.

67. Ellen J. Ravndal, ‘“A Force for Peace”: Expanding the Role of the UN Secretary-General under Trygve Lie, 1946–1953’, *Global Governance*, 23(3), 2017, pp. 443–59.

68. Trygve Lie, ‘2nd Inaugural Speech’ A/PV.299 (United Nations General Assembly, 1950), available at: http://www.un.org/ga/search/view_doc.asp?symbol=A/PV.299; Trygve Lie, *In the Cause of Peace: Seven Years* w*ith the United Nations* (New York: The Macmillan Company, 1954), p. 342.

69. Peter Wallensteen, ‘Dag Hammarskjöld’s Diplomacy: Lessons Learned’, in Carsten Stahn and Henning Melber (eds) *Peace Diplomacy, Global Justice and International Agency: Rethinking Human Security and Ethics in the Spirit of Dag Hammarskjöld* (Cambridge: Cambridge University Press, 2014), pp. 385–6.

70. A. W. Dorn and Robert Pauk, ‘Unsung Mediator: U Thant and the Cuban Missile Crisis’, *Diplomatic History*, 33(2), 2009, pp. 261–292.

71. Anthony F. Lang, ‘A Realist in the Utopian City: Boutros Boutros-Ghali’s Ethical Framework and Its Impact’, in Kent J. Kille (ed.) *The UN Secretary-General and Moral Authority*, pp. 265–297; Simon Rushton, ‘The UN Secretary-General and Norm Entrepreneurship: Boutros Boutros-Ghali and Democracy Promotion’, *Global Governance*, 14, 2008, pp. 95–110.

72. Thomas G. Weiss and Tatiana Carayannis, ‘Windows of Opportunity for UN Reform: Historical Insights for the Next Secretary-General’, *International Affairs*, 93(2), 2017, pp. 316–9.

73. António Guterres, ‘Conclusion: A Beacon of Inspiration for the Family of Nations’, in Silvano M. Tomasi (ed.) *The Vatican in the Family of Nations: Diplomatic Actions of the Holy See at the UN and Other International Organizations in Geneva* (Cambridge: Cambridge University Press, 2017), pp. 829–849.

74. Rushton, ‘The UN Secretary-General and Norm Entrepreneurship’; Kent J. Kille, ‘The Secular Pope: Insights on the UN Secretary-General and Moral Authority’, in Kent J. Kille (ed.) *The UN Secretary-General and Moral Authority*, pp. 337–354.

75. Inis L. Claude, *Swords Into Plowshares: The Problems and Progress of International Organization*, 4th ed. (New York: Random House, 1971), pp. 207–8; Michael B. Schroeder, ‘Executive Leadership in the Study of International Organization: A Framework for Analysis’, *International Studies Review*, 16(3), 2014, pp. 339–361; Robert W. Cox, ‘The Executive Head: An Essay on Leadership in International Organization’, *International Organization*, 23(2), 1969, pp. 205–30; Mark Menaldo, *Leadership and Transformative Ambition in International Relations* (Cheltenham: Edward Elgar, 2013), p. 172; Nina Hall and Ngaire Woods, ‘Theorizing the Role of Executive Heads in International Organizations’, *European Journal of International Relations*, 24(4), 2018, pp. 865–86.

76. Claude, *Swords Into Plowshares*, p. 211.

77. Lipsey, *Hammarskjold*, p. 153.

78. Anne Stensvold, ‘Religion, State and Symbol Politic: The Catholic Church at the UN’, in Anne Stensvold (ed.) *Religion, State and the United Nations: Value Politics* (London: Routledge, 2017), p. 95.

79. Leon Gordenker, ‘U Thant and the Office of U.N. Secretary-General’, *International Journal*, 22(1), 1967, p. 6; Jodok Troy, ‘Two “Popes” to Speak for the World: The Pope and the United Nations Secretary General in World Politics’, *The Review of Faith & International Affairs*, 15(4), 2017, pp. 67–78.

80. Ramesh Chandra Thakur, *The United Nations, Peace and Security: From Collective Security to the Responsibility to Protect* (Cambridge: Cambridge University Press, 2006), p. 333; Ramesh C. Thakur, ‘Choosing the Ninth United Nations Secretary-General: Looking Back, Looking Ahead’, *Global Governance*, 23, 2017, p. 3.

81. Javier P. de Cuéllar, ‘Reflecting on the Past and Contemplating the Future’, *Global Governance*, 1, 1995, p. 168.

82. Kusumita P. Pedersen, ‘Sri Chinmoy’s Work at the United Nations: Spirituality and the Power of Silence’, *CrossCurrents*, 60, 2010, p. 339.

83. Kent J. Kille (ed.), *The UN Secretary-General and Moral Authority: Ethics and Religion in International Leadership* (Washington, DC: Georgetown University Press, 2007).

84. Menaldo, *Leadership and Transformative Ambition in International Relations*, p. 172.

85. John Mathiason, ‘What Kind of International Public Service Do We Need for the Twenty-First Century?’, *Global Governance*, 14(2), 2008, p. 128.

86. Cuéllar, ‘Reflecting on the Past and Contemplating the Future’, p. 168.

87. Kokosalakis, ‘Legitimation Power and Religion in Modern Society’, p. 368.

88. Kokosalakis, ‘Legitimation Power and Religion in Modern Society’, p. 372.

89. Hurd, ‘Enchanted and Disenchanted International Law’, p. 96.

90. See also Rebecca Sanders, *Plausible Legality: Legal Culture and Political Imperative in the Global War on Terror* (New York: Oxford University Press, 2018).

91. Hurd, ‘Enchanted and Disenchanted International Law’, p. 98.

92. Kokosalakis, ‘Legitimation Power and Religion in Modern Society’, p. 371.

93. Oscar Schachter, ‘Human Dignity as a Normative Concept’, *The American Journal of International Law*, 77(4), 1983, p. 853.

94. The ‘offhandedness of the decision to place it early in the preamble and the absence of any consideration of its significance argue against inferring any shared, articulate purpose’. Charles R. Beitz, ‘Human Dignity in the Theory of Human Rights: Nothing but a Phrase?’, *Philosophy & Public Affairs*, 41(3), 2013, p. 266; see also Samuel Moyn, *Human Rights and the Uses of History*, Expanded new edition (London: Verso, 2017), p. 28.

95. Schachter, ‘Human Dignity as a Normative Concept’, p. 853.

96. Mazower, *Governing the World*, p. 380; Anne Orford, *International Authority and the Responsibility to Protect* (Cambridge: Cambridge University Press, 2011).

97. The ‘third UN’ consists of ‘nongovernmental organizations (NGOs), external experts, scholars, consultants, and committed citizens who work closely with the UN’s intergovernmental machinery and secretariats’. Weiss et al., ‘The “Third” United Nations’, p. 123.

98. For example, Michael Barnett and Janice Gross Stein, *Sacred Aid: Faith and Humanitarianism* (Oxford: Oxford University Press, 2012); Stephen Hopgood, *Keepers of the Flame: Understanding Amnesty International* (Ithaca, NY: Cornell University Press, 2006).

99. Gili S. Drori, ‘United Nations’ Dedications’, *International Sociology*, 20(2), 2005, p. 190.

100. Rieff, *A Bed for the Night*, p. 15.

101. Ban Ki-Moon, ‘One Humanity: Shared Responsibility’, A/70/709 (Report of the Secretary-General for the World Humanitarian Summit, United Nations, 2016), para. 27.

102. Schachter, ‘Human Dignity as a Normative Concept’, p. 851.

103. Schachter, ‘Human Dignity as a Normative Concept’, p. 854. On the issues of migrants, for example, Ki-Moon grounds the legitimation for humanitarian assistance and political solutions in a turn towards an individual identification with the refugee: Their ‘concern’ and ‘daily struggles’ must become ours. Ki-Moon, ‘One humanity’, para. 19.

104. Voeten, ‘The Political Origins of the UN Security Council’s Ability to Legitimize the Use of Force’, p. 551. Hans Morgenthau and Raymond Aron, for instance, pointed out at the example of the evolution of the European Community that functional elements and economic growth are too less to hold people and different interests in any such community together. Hans J. Morgenthau, *Politics Among Nations: The Struggle for Power and Peace*, 5th ed. (New York: Alfred A. Knopf, 1973), pp. 513–19. Brian C. Anderson, *Raymond Aron: The Recovery of the Political* (Lanham, MD: Rowman & Littlefield, 1998), p. 177. See also Ramesh Thakur, ‘Law, Legitimacy and the United Nations’, in Richard A. Falk, Mark Juergensmeyer and Vesselin Popovski (eds) *Legality and Legitimacy in Global Affairs* (Oxford: Oxford University Press, 2012).

105. Claude, ‘Collective Legitimization as a Political Function of the United Nations’, p. 368. Franck, *The Power of Legitimacy Among Nations*; David P. Rapkin and D.A.N. Braaten, ‘Conceptualizing Hegemonic Legitimacy’, *Review of International Studies*, 35(1), 2009, 113–49.

106. Alison McQueen, *Political Realism in Apocalyptic Times* (Cambridge: Cambridge University Press, 2018), pp. 147–191.

107. Rieff, *A Bed for the Night*, p. 156.

108. United Nations, Press Release SG/382: Address by Secretary-General Dag Hammarskjöld at University of California, Convocation Berkeley, California (1954), https://goo.gl/gFuQfn, p. 7. The first acting Secretary-General of the UN, Jebb Gladwyn, took a similar stand. Gladwyn, *The Memoirs of Lord Gladwyn*, p. 257.

109. Foote, *Dag Hammarskjöld*, p. 319.

